# The pain receptor TRPV1 displays agonist-dependent activation stoichiometry

**DOI:** 10.1038/srep12278

**Published:** 2015-07-21

**Authors:** Adina Hazan, Rakesh Kumar, Henry Matzner, Avi Priel

**Affiliations:** 1 The Institute for Drug Research (IDR), School of Pharmacy, Faculty of Medicine, The Hebrew University of Jerusalem, Jerusalem 91120, Israel

## Abstract

The receptor channel TRPV1 (Transient Receptor Potential Vanilloid 1) is expressed by primary afferent sensory neurons of the pain pathway, where it functions as a sensor of noxious heat and various chemicals, including eicosanoids, capsaicin, protons and peptide toxins. Comprised of four identical subunits that organize into a non-selective cationic permeable channel, this receptor has a variety of binding sites responsible for detecting their respective agonists. Although its physiological role as a chemosensor has been described in detail, the stoichiometry of TRPV1 activation by its different ligands remains unknown. Here, we combined the use of concatemeric constructs harboring mutated binding sites with patch-clamp recordings in order to determine the stoichiometry for TRPV1 activation through the vanilloid binding site and the outer-pore domain by capsaicin and protons, respectively. We show that, while a single capsaicin-bound subunit was sufficient to achieve a maximal open-channel lifetime, all four proton-binding sites were required. Thus, our results demonstrate a distinct stoichiometry of TRPV1 activation through two of its different agonist-binding domains.

The pain receptor channel TRPV1 (Transient Receptor Potential Vanilloid 1), the ‘heat and capsaicin receptor’, plays a key role in the detection of a large array of noxious stimuli^1^,^2^. TRPV1 is expressed by primary afferent sensory neurons of the pain pathway, where it functions as a sensor of noxious heat (>42 °C) and various noxious chemicals, including eicosanoids, capsaicin (the active component of chili peppers), protons and peptide toxins^3^,^4^,^5^. TRPV1’s overall transmembrane topology and subunit organization, illustrated in its recently solved cryo-EM structures, resembles voltage-gated potassium (K_V_) channels, where a functional channel is formed by the symmetrical arrangement of four identical subunits around a central ion-conducting pore^6^,^7^. Although its physiological role as a chemosensor has been described in detail, the molecular mechanism underlying its unique ability to respond to various types of agonists is not fully understood.

The TRPV1 channel displays distinct putative binding sites for its corresponding agonists^8^,^9^,^10^,^11^,^12^,^13^,^14^,^15^. For example, capsaicin, which is a small hydrophobic molecule, binds to the intracellulary-located vanilloid binding site (VBS), while protons bind to the extracellular outer-pore domain^8^,^9^. Interestingly, while these binding sites are located in physically separate regions throughout the receptor channel subunits, they both elicit channel activation^16^. Whether the diverse ligand-binding domains govern TRPV1 activation through similar or different allosteric mechanisms remains largely unknown. Furthermore, the stoichiometry of TRPV1 activation by its different ligands is yet to be described. Elucidating TRPV1 activation mechanism(s) would uncover the molecular basis of this receptor’s unique ability to respond to a large array of noxious chemical stimuli.

Here, we determined the stoichiometry for activation of TRPV1 through the VBS and the outer-pore domain by capsaicin and protons, respectively. By engineering concatemeric TRPV1 constructs harboring various combinations of wild-type and mutated subunits, we established the minimal number of capsaicin- and proton- bound sites required to evoke channel activation. Our results indicated that, while a single capsaicin-bound subunit is sufficient to achieve a maximal open-channel lifetime, all four proton-binding sites are required. We therefore propose that the two studied TRPV1 agonists activate the channel via distinct mechanisms, indicating a ligand-dependent stoichiometry of TRPV1 activation.

## Results

### Concatemeric TRPV1 is self-assembled and mimics wild type activation pattern

**Figure 1:**
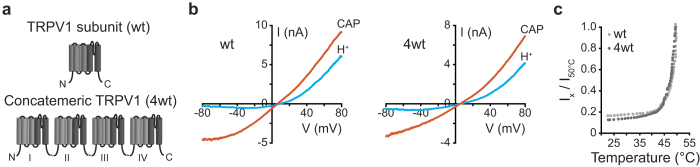
TRPV1 tetrameric concatemer mimics the activation profile of the wild-type protein. (**a**) Schematic representation of wt and 4wt TRPV1 constructs. The concatemeric rat TRPV1 (rTRPV1) construct was engineered by insertion of a unique restriction enzyme site at the C terminal (without the stop codon) and to the N-terminal (without the start codon) of adjacent wt subunits (bottom; dashed line). (**b**) Both capsaicin (CAP; 1 μM; orange line) and extracellular protons (H^+^; pH 5.5; cyan line) elicited robust, outwardly rectifying currents in HEK293 cells transiently expressing either rTRPV1 (“wt”; left) or concatemeric rTRPV1 (four subunits, “4wt”; right) construct. Current-voltage relationship traces were recorded using whole-cell patch-clamp recording (in 1 s^−1^ voltage-ramps between −80 and +80 mV). (**c**) Thermal response profiles of oocytes expressing either rTRPV1 (“wt”; light grey) or concatemeric rTRPV1 (“4wt”; dark grey) construct. The current at each indicated temperature was normalized to that evoked at 50 °C. Temperature threshold for both wt and 4wt constructs was 43 °C; the Q_10_ was 27 for wt and 26 for 4wt rTRPV1.

In order to determine the stoichiometry for activation of TRPV1 through the VBS and the outer-pore domain, we generated concatemeric TRPV1 constructs. Concatemers have been proven a useful tool to control the number and location of a mutated subunit of a homomeric protein^17^,^18^,^19^,^20^,^21^,^22^,^23^. TRPV1 is assembled as a homotetrameric channel with intracellular N- and C-termini^6^,^24^. Using this feature as guidelines, we generated concatemeric wild type (wt) rat TRPV1 (rTRPV1) constructs (illustrated in Fig. 1a).

**Figure 2:**
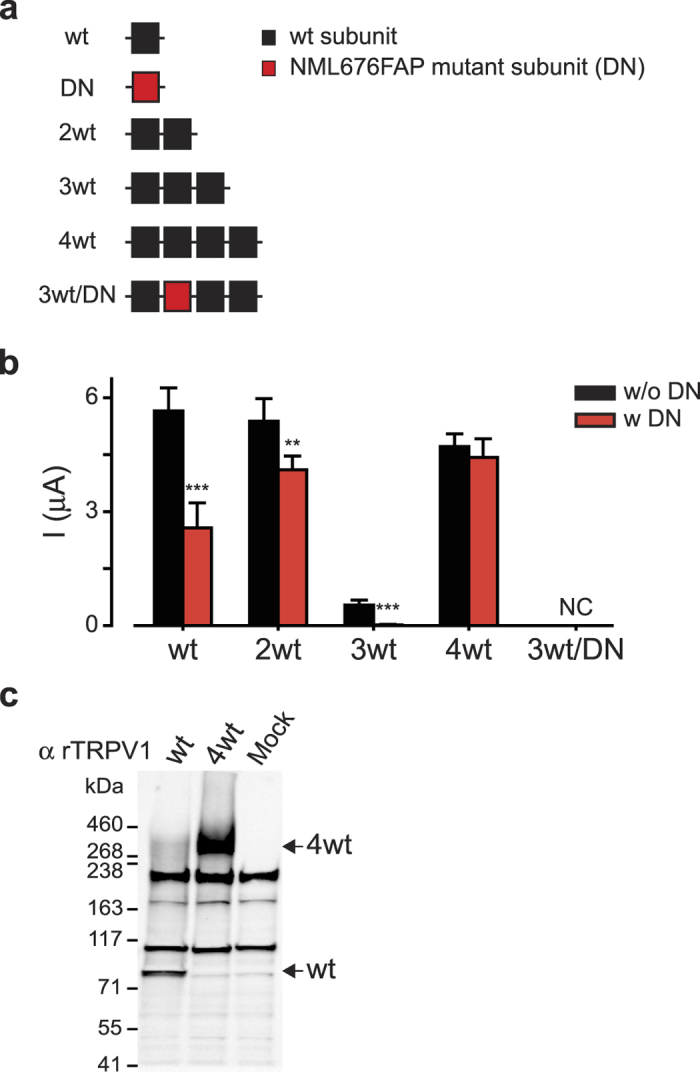
TRPV1 tetrameric concatemer self-assembles into a functional receptor channel. (**a**) Schematic illustration of the different tandem constructs used to study the assembly of rTRPV1 concatemers. Black squares represent wild type rTRPV1 subunit (wt) and red squares represent dominate-negative subunits (DN) mutated in positions NML676FAP. (**b**) Bar diagram represents the average (±SEM) amplitudes of a whole-cell, capsaicin-evoked (1 μM) currents from *Xenopus laevis* oocytes expressing the different tandem constructs either alone (black bars), or together with a DN construct (red bars; DN). Holding potential −60 mV. Each bar represents readings from 6–15 oocytes. The statistical significance between currents evoked with or without DN was determined using unpaired Student’s *t* tests, where **represents P ≤ 0.01, ***represents P ≤ 0.001. Note that no statistical significant difference in evoked currents was found between cells expressing 4wt with or without DN. (**c**) Western-blot analysis of HEK293T cells transiently expressing a single subunit (wt) or a tetrameric tandem (4wt) constructs, using an anti-rat TRPV1 antibody (α rTRPV1). Note that, while the expression of a single subunit wt construct yielded a band corresponded to ~90 kDa, the only specific band observed in cells expressing the 4wt construct corresponded to a ~400 kDa size (indicated with arrows).

To verify the functionality of the tetrameric TRPV1 concatemer, we analyzed its response to its known agonists: capsaicin, protons and heat (Fig. 1)^19^,^25^. HEK293T cells transiently expressing the native rTRPV1 (wt) or tetrameric rTRPV1 concatemeric construct (four subunits, “4wt”) were continuously perfused with the standard extracellular solution when protons (pH 5.5) were applied for 15 s, followed by a minute wash, and finally capsaicin (1 μM). To avoid contamination and due to its hydrophobic nature, applications of high capsaicin concentrations (>0.2 μM) were restricted to the end of the experiments throughout the study. The current-voltage relationship was determined using whole-cell patch-clamp recordings. As shown in Fig. 1b, both extracellular protons (H^+^; cyan line) and capsaicin (CAP; orange line) elicited robust, outwardly rectifying currents in cells expressing either rTRPV1 (wt) or tetrameric rTRPV1 concatemer (4wt) construct. We further determined the current-voltage relationships in response to heat (temperature range of 22^°^C–50^°^C) or capsaicin by applying the two electrodes voltage clamp (TEVC) technique in *Xenopus laevis* oocytes expressing wt or 4wt concatemer (Figs 1c and 2; average currents at +80 mV). In both heterologous systems expressing the 4wt concatemeric receptor, the current amplitude and kinetics were comparable to those previously reported for wt rTRPV1 (average current amplitude (±SEM): HEK293T: wt I_CAP+80mV_ = 8.3 ± 1.2 nA and 4wt I_CAP+80mV_ = 7.8 ± 1.8 nA; oocytes: wt I_CAP+80mV_ = 5.6 ± 0.3 μA and 4wt I_CAP+80mV_ = 4.7 ± 0.2 μA; average temperature threshold (±SEM): wt: 42 ± 1.2 °C; 4wt: 43 ± 1.6 °C, and average temperature sensitivity (±SEM): wt: Q_10_ = 27 ± 3; 4wt: Q_10_ = 25 ± 2; Figs 1 and 2)^10^,^12^,^26^,^27^,^28^.

A potential pitfall in using concatemeric constructs is the possibility of the assembly of a functional channel from multiple polypeptides, which would result in a mixed channel population in the plasma membrane^18^,^19^. To test for this possibility, we generated various concatemeric wt rTRPV1 constructs, ranging in length from dimeric (2wt), trimeric (3wt), to tetrameric (4wt) and co-expressed them with the previously described dominant-negative (DN) rTRPV1 (NML676FAP) subunit (Fig. 2a)^29^. In order to retain a known cRNA molecular ratio between the concatemeric constructs and the DN subunit, oocytes were used for this analysis. As shown in Fig. 2b, the presence of DN rTRPV1 rendered significant reduction in the current elicited in response to capsaicin in wt, 2wt and 3wt constructs, while the 4wt current remained unaffected. Thus, the wt, 2wt and 3wt constructs formed a channel consisting of subunits from multiple polypeptides, while the tetrameric (4wt) concatemer assembled into a channel consisting of a single polypeptide. To further validate this finding, we generated a tetrameric concatemer in which the second subunit was mutated to the DN form (3wt/DN; wt-DN-wt-wt; Fig. 2a), and analyzed its response to heat-, proton- or capsaicin. We could not observe agonist-evoked currents as late as 10 days after construct injection to the oocytes (Fig. 2b). Next, we ruled out cleavage of the constructs in the process of assembly and trafficking by performing western blot analysis for rTRPV1 of the single subunit (wt) and the concatemeric construct (4wt)(Fig. 2c). Taken together, these findings demonstrate self-assembly of the 4wt rTRPV1 polypeptide into a functional channel.

**Figure 3:**
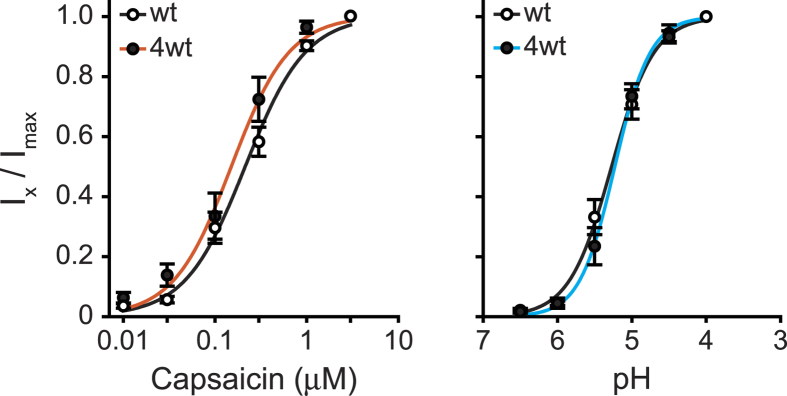
TRPV1 tetrameric concatemer maintains sensitivity to capsaicin and protons. Normalized concentration-response relationships for capsaicin (left) and protons (right) of rTRPV1 (wt; empty circles) and the tetrameric concatemeric construct (4wt; full circles) transiently expressed in HEK293 cells. Each point represents the average (±SEM) response of 6–12 cells. Solid lines (black for both agonists of wt; orange for capsaicin, and cyan for protons of 4wt) are fit to the Hill equation (see Eq. (2)) with EC_50_ and n_H_ for capsaicin of 0.24 ± 0.02 μM and 1.3 for the wt receptor and 0.15 ± 0.02 μM and 1.4 for the 4wt construct, respectively. The EC_50_ and n_H_ for protons are pH 5.27 ± 0.28 and 1.5 for the wt receptor and pH 5.23 ± 0.24 and 1.8 for the 4wt construct, respectively.

In order to verify that our tetrameric construct maintained the sensitivity of the receptor channel to its different activators, we tested capsaicin and protons concentration-dependent activation of wt and 4wt rTRPV1 (Fig. 3). Using the whole-cell configuration of the patch clamp technique at a holding potential of −40 mV, each cell was exposed to increasing concentrations of the activator (capsaicin or protons) with a 20–30 s wash between each application, when each activator was analyzed separately. Based on the concentration-response relationship fitted by the Hill equation for the effect of capsaicin and protons (Fig. 3), the estimated EC_50_ and Hill coefficient (n) for the 4wt TRPV1 construct were comparable to these estimated for the wt construct (for capsaicin: EC_50_ = 0.24 ± 0.02 μM and n_H_ = 1.3 for wt and EC_50_ = 0.15 ± 0.02 μM and n_H_ = 1.4 for 4wt; For protons: EC_50_ = pH 5.27 ± 0.28 and n_H_ = 1.5 for wt and EC_50_ = pH 5.23 ± 0.24 and n_H_ = 1.8 for 4wt; Fig. 3). Therefore, our results indicate that the tetrameric concatemeric construct mimics the activation pattern and agonist sensitivity of TRPV1 to its different activators.

### A single available vanilloid binding site (VBS) is sufficient to activate TRPV1

**Figure 4:**
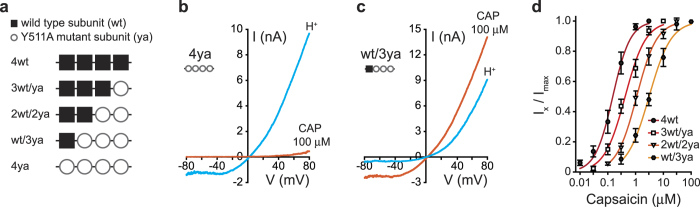
A single VBS-containing subunit is sufficient to evoke capsaicin sensitive current. (**a**) Schematic representation of the different tetrameric concatemeric constructs used to study the stoichiometry for TRPV1 activation by capsaicin. Black squares represent wt subunits while empty circles represent subunits with an Y511A mutated VBS (ya). (**b, c**) Current-voltage relationship traces in HEK293 cells transiently expressing the 4ya (b) and wt/3ya (c) constructs in response to capsaicin (CAP; 100 μM; orange line) and protons (H^+^; pH 5.5; cyan line). Currents were recorded using whole-cell patch-clamp recording (in 1 s^−1^ voltage ramps between −80 and +80 mV). Mutations in all VBS nearly eliminated the capsaicin response, while the proton response was intact (b). A single intact VBS was sufficient to produce a robust capsaicin response (c). (**d**) Normalized concentration-response relationships for capsaicin of the different concatemers. Each point represents the average (±SEM) response of 10–14 HEK293 cells transiently expressing the respective construct. Solid lines are fit to the Hill equation (see Eq. (2)): 4wt (full circles, dark red line; n_H_ = 1.4; EC_50_ = 0.15 ± 0.02 μM), 3wt/ya (empty squares, red line; n_H_ = 1.1; EC_50_ = 0.42 ± 0.07 μM), 2wt/2ya (empty triangle; orange line; n_H_ = 1.2; EC_50_ = 1.09 ± 0.08 μM) and wt/3ya (full diamonds, yellow line; n_H_ = 1.1; EC_50_ = 3.10 ± 0.47 μM). Reduction in the number of subunits containing an intact VBS leads to a shift in the affinity of capsaicin.

Activation of TRPV1 by exo-vanilloids (such as capsaicin) and endo-vanilloids (such as 12-(S)- and 15-(S)-hydroperoxyeicosatetraenoic acid (12- and 15-HPETE)) has been studied extensively in order to better understand pain physiology and behavior^5^,^25^,^30^; however, the stoichiometry and allosteric regulatory mechanisms through which they activate TRPV1 remain unknown. The Y511A (YA) substitution, located in the vanilloid binding site (VBS), was shown by mutagenesis and biochemical studies to substantially decrease the sensitivity of TRPV1 to capsaicin^9^,^31^,^32^,^33^. In order to determine the number of subunits necessary for capsaicin-mediated TRPV1 activation, we generated a series of concatemeric constructs composed of subunits that harbor either a wt (wt) or a mutated VBS (containing the YA substitution; ya) in various subunit combinations (Fig. 4a). We transiently expressed different concatemeric constructs containing various ratios of wt and mutated subunits in HEK293T cells and recorded the currents evoked by capsaicin and protons using whole-cell recordings. As expected, the 4ya construct (in which VBS is mutated in all subunits) was insensitive to capsaicin, even when the agonist was applied at a maximal concentration (100 μM due to solubility; three orders of magnitude above the EC_50_ of the wt and 4wt, Fig. 3), while maintaining its sensitivity to protons (Fig. 4b). Interestingly, the inclusion of a single wt subunit (wt/3ya) was sufficient to induce a robust outward-rectifying current in response to a high capsaicin concentration (100 μM; Fig. 4c). Therefore, our results indicate that a single VBS subunit is sufficient to activate the channel.

Next, we determined the concentration-response relationship of the various concatemeric constructs, using the whole-cell configuration of the patch clamp at holding voltage of −40 mV. As shown in Fig. 4d, although elimination of capsaicin binding sites decreases receptor sensitivity to its agonist (4wt: EC_50_ = 0.15 μM while wt/3ya: EC_50_ = 3.10 μM), the Hill coefficient was unaffected and remained close to the values obtained for the 4wt and wt TRPV1 (wt: n_H_ = 1.3; 4wt: n_H_ = 1.4; 3wt/ya: n_H_ = 1.1; 2wt/2ya: n_H_ = 1.2; wt/3ya: n_H_ = 1.1). Thus, these results suggest that a single VBS-containing subunit is sufficient to produce the conformational changes required for channel activation, indicating that the symmetry model (MWC) best describes the allosteric regulation of TRPV1 in response to capsaicin^20^,^22^,^34^,^35^. Moreover, the high affinity of capsaicin to TRPV1 is possibly an outcome of its ability to bind each one of the four binding sites independently, as evident by the gradual change in the sensitivity in direct relation to the number of available intact VBS.

**Figure 5:**
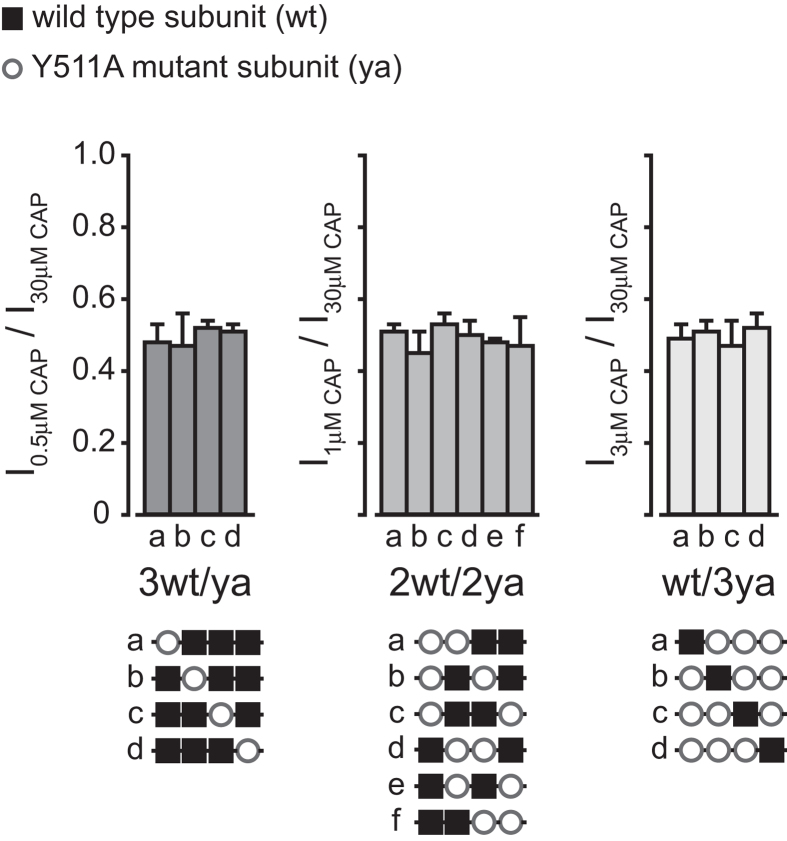
Activation of TRPV1 by capsaicin displays channel symmetry. Bar diagram representing the average (±SEM) amplitude of the whole-cell currents for each indicated group of tandem constructs evoked by the relative capsaicin concentration (3wt/ya group: 0.5 μM, left bars; 2wt/2ya group: 1 μM, middle bars; wt/3ya group: 3 μM, right bars) normalized to the current amplitude of 30 μM capsaicin (I_30μM CAP_). Tested conditions were used according to the dose response determined in Fig. 4d. Below each bar diagram are schematic Illustrations of the different tandem constructs used to study the symmetry of the capsaicin response. Black squares represent wt subunits (wt) and empty circles represent subunits with a mutated VBS in position Y511A (ya). Holding potential −40 mV. Each bar represents 4–7 HEK293T cells transiently expressing the respective construct. Statistical significance was determined with the unpaired Student’s *t* tests. No statically significant differences were found.

In order to determine whether the arrangement of intact and mutated subunit(s) affects channel activity, we generated all the tetrameric concatemer combinations for the 3wt/ya, wt/3ya and the 2wt/2ya, and analyzed their sensitivity to capsaicin by whole cell recordings (Fig. 5). If the location of the mutated subunit(s) affects channel sensitivity, the various combinations within each group are expected to have different response. As shown in Fig. 5, within each group, the different constructs showed comparable sensitivity to capsaicin, regardless of the location of the mutated subunit(s), demonstrating symmetry of the channel.

### The open probability of TRPV1 is affected by the number of active VBS

**Figure 6:**
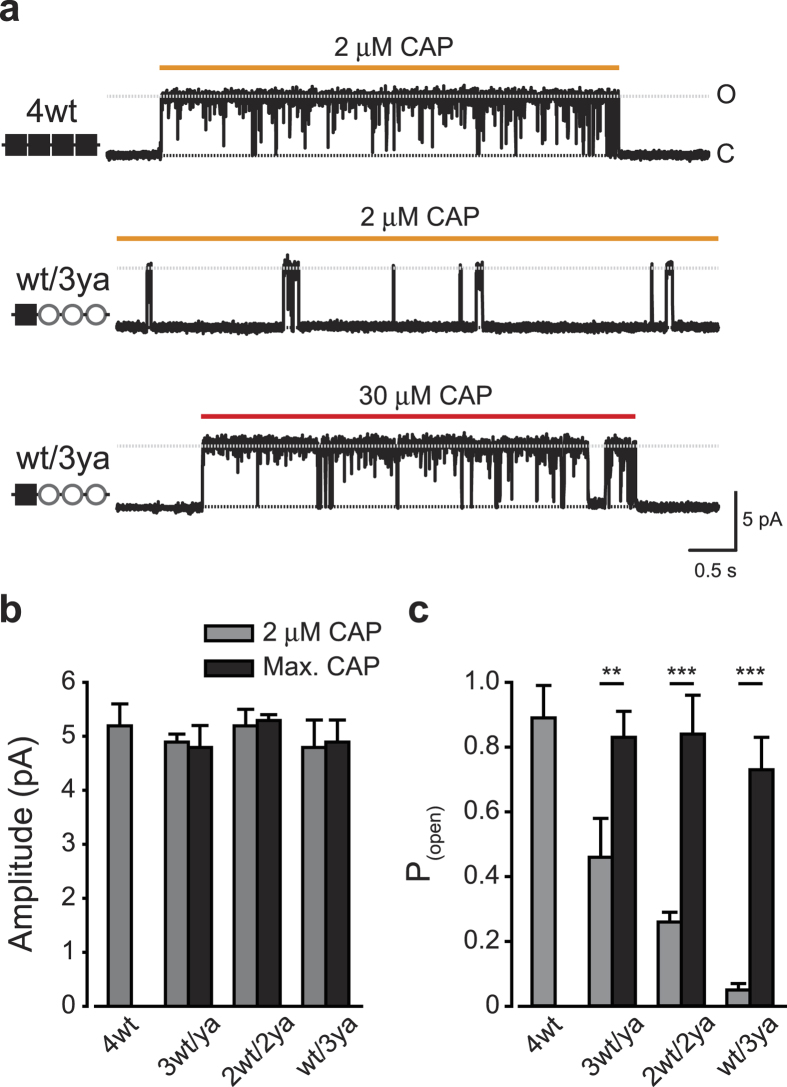
A single VBS bound subunit evokes full channel activation. (**a**) Representative current records from an excised outside-out membrane patch of capsaicin-exposed Flp-in TREX HEK293 cells stably transfected with 4wt and wt/3ya TRPV1 concatemers. Upward (outward) currents indicate channel opening (grey dash line). Shown are representative channel activities upon exposing the patches to saturating capsaicin concentration (2 μM CAP; orange line) of 4wt (top trace) and wt/3ya (middle trace) concatemers. Also shown is a representative channel activity of the same wt/3ya patch exposed to 30 μM capsaicin (30 μM CAP; red line; bottom trace). Holding potential at +60 mV sampled at 50 kHz and filtered at 1 kHz for display. Note the similar channel activation for the relative saturating capsaicin concentration of the two constructs. (**b**, **c**) Bar diagram representing the average (±SEM) amplitude (**b**) and open probability (P_(open)_; (**c**) of the single-channel current activated by capsaicin at 2 μM (grey bars) and saturating concentration (Max. CAP; black bars) of the various concatemeric constructs. Each bar represents an average of 3–5 patches. The statistical significance between the 2 μM and the relative saturating concentration of capsaicin was determined using paired Student’s *t* test, where **represents *P* ≤ 0.01 and ***represents *P* ≤ 0.001. Note that all the different VBS-mutated concatemeric constructs reached similar open probability to that reached by the 4wt construct at saturating capsaicin concentrations.

Our results are consistent with a concert model of capsaicin-mediated TRPV1 activation. In order to uncover the mechanisms by which the sensitivity to capsaicin increases in direct relation to the number of VBS-containing subunits (Fig. 4d), single-channel currents were measured in the outside-out configuration of the patch-clamp technique (Fig. 6)^36^. In order to facilitate recording from a single channel, we used an inducible system, Flp-in TREX, which allows doxycycline-controlled construct expression levels. This system was shown to be highly useful in obtaining membrane patches with a single channel, which is challenging to achieve in transiently transfected cells due to high TRPV1 expression levels^12^,^37^. We therefore stably transfected Flp-in TREX HEK293 cells with the capsaicin sensitive concatemeric constructs for single channel analyses. The membrane potential was held at +60 mV considering that the conductance of TRPV1 receptor channels is higher at positive potentials^12^,^38^,^39^. The pipette contained standard intracellular patch solution, and the patch of membrane was continuously perfused with standard extracellular solution. First, we selected outside out patches containing a functional, single channel by exposing them to capsaicin (2–30 μM). Bursts of single channel activity were detected in 74 of 354 (~21%) tested patches, but, in 58 of them, channel activity was lost within 20 s and did not recover following several minutes of wash; therefore, we used the remaining 16 single channels for further analysis. Representative current traces recorded in the presence of 2 μM capsaicin for 4wt and wt/3ya channels are shown in Fig. 6a (top traces). In the 4wt receptor at 2 μM capsaicin (saturated concentration for this construct; Fig. 3), a flickering burst of activity was evoked with an average amplitude of 5.2 ± 0.4 pA (corresponding to unitary conductance of 86 ± 3 pS) and open probability of 0.89 ± 0.10 (Fig. 6a, top trace). However, in the presence of only a single wt subunit (wt/3ya), the same capsaicin concentration evoked short openings with similar average amplitude of 4.8 ± 0.5 pA (corresponding to unitary conductance of 80 ± 5 pS) but with an open probability of 0.05 ± 0.02 (Fig. 6a, middle trace). When we exposed the wt/3ya construct patch to 30 μM capsaicin (a saturating concentration for this construct; Fig. 4d), flickering bursts of activity was observed, with an average amplitude of 4.9 ± 0.4 pA (corresponding to unitary conductance of 82 ± 3 pS) and an open probability of 0.73 ± 0.10 (Fig. 6a, bottom trace). Fig. 6b,c summarize the average amplitude and the open probability of the analyzed capsaicin sensitive constructs in 2 μM and the relative saturating capsaicin concentration (as determined in Fig. 4d). Our results show that, while the unitary conductance remains same regardless of agonist concentration (Fig. 6b), the open probability was strongly correlated with the number of putative subunits (at 2 μM; Fig. 6c, grey bars). Nevertheless, at relative saturating concentration for each of the constructs, similar high open probability bursts of activity were obtained. Thus, our results indicate that a single active VBS is sufficient to achieve a maximal open-channel lifetime.

### A maximal proton-mediated TRPV1 activation requires all four subunits

**Figure 7:**
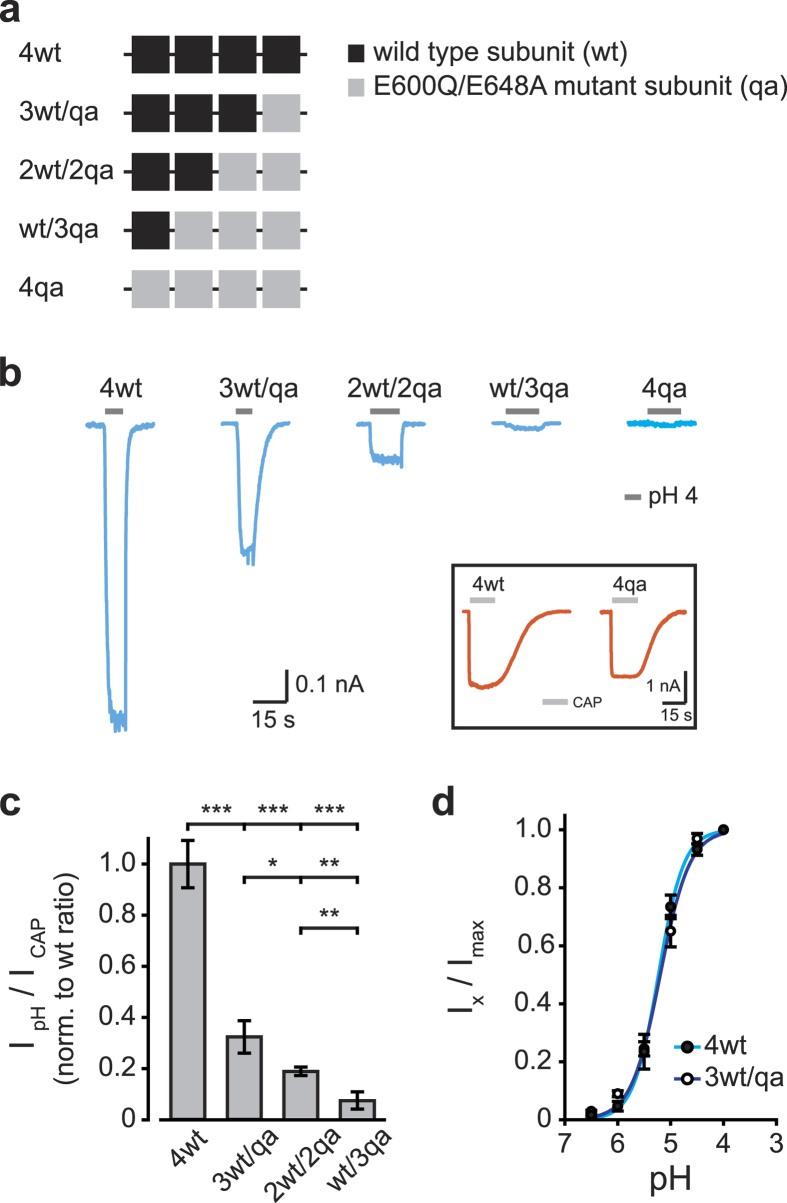
TRPV1 maximal activation by protons requires all four proton-binding subunits. (**a**) Illustration of the different tetrameric concatemeric constructs used to study the stoichiometry for TRPV1 activation by protons. Black squares represent proton-binding intact subunits (wt) and grey squares represent subunits mutated in their proton-binding site in both positions E600Q and E648A (qa). (**b**) Whole-cell current traces (blue) in response to protons (pH 4; grey bar) of HEK293T cells transiently expressing the different mutant protons-binding concatemeric constructs, as indicated. Similar TRPV1 expression levels were estimated from the current evoked by saturating capsaicin concentration. *Inset:* representative whole-cell current traces (orange) in response to capsaicin (1 μM; light grey bar) of the 4wt and 4qa constructs. Note that the capsaicin response was unaffected by the mutation in the proton-binding sites. Holding potential of −40 mV. (**c**) Bar diagram representing the average ratio (±SEM) between protons (pH 4) - and capsaicin (at saturating concentration; 1 μM) - evoked amplitudes, normalized to the ratio obtained for the 4wt construct. Each bar represents 12–14 HEK293T cells transiently expressing the indicated concatemeric constructs. The statistical significance between the 4wt and the different proton-binding mutant constructs was determined with unpaired Student’s *t* test, where * represents *P* ≤ 0.05, **represents *P* ≤ 0.01 and ***represents *P* ≤ 0.001. Note a reduction in protons to capsaicin ratio with the inclusion of a single subunit mutated in its proton-binding sites (3wt/qa). (**d**) Normalized concentration-response relationships for protons of the 4wt and 3wt/qa concatemeric constructs. Each point represents the average (±SEM) response of 5–8 HEK293T cells transiently expressing the indicated concatemeric constructs. Solid lines are fit to the Hill equation (see Eq. (2)): 4wt (full circles, cyan line; n_H_ = 1.8; EC_50_ = pH 5.23 ± 0.24), 3wt/qa (empty circle, blue line; n_H_ = 1.6; EC_50_ = pH 5.20 ± 0.32). Holding potential of −40 mV.

To determine TRPV1 activation mechanism through an alternative ligand binding site, we analyzed the subunit stoichiometry of proton activation. The E600Q (EQ) and E648A (EA) substitutions located on the outer-pore domain of rTRPV1 substantially decrease its activation by low pH^8^,^40^,^41^,^42^. In order to assess the proton-mediated TRPV1 activation mechanism, we generated a series of concatemeric constructs containing subunits that harbor either an intact or a mutated (containing the E600Q/E648A dual substitutions; qa) protons binding site (Fig. 7a). We recorded the currents evoked by protons and capsaicin from HEK293T cells transiently expressing the different constructs using the whole-cell configuration of the patch clamp technique (Fig. 7b). The analyzed cell was continuously perfused with the standard extracellular solution. Concurrently, the cell was exposed initially to protons (pH 4) for 15 s, followed by minute wash, and finally capsaicin (1 μM). A high proton concentration (pH 4) was used in order to saturate all binding sites, while higher proton concentrations (pH < 4) were excluded from analysis due to patch instability and leak currents. Throughout all the experiments, the extracellular solution and bath solution were supplemented with 50 μM Amiloride to block ASIC1a activity, which is endogenously expressed by HEK293T cells^12^,^42^,^43^. Membrane currents in response to protons (pH 4) were observed in concatemeric constructs having at least one proton-binding subunit (4wt, wt/3qa, 2wt/2qa and 3wt/qa; Fig. 7b). Representative traces shown in Fig. 7b were extracted from cells with comparable expression level, as estimated by the current amplitude for saturating capsaicin concentration (1 μM, 1.5-1.8 nA at −40 mV; see inset). The ratio between proton (pH 4) - and capsaicin (1 μM) - mediated activation of the different constructs was normalized to the protons/capsaicin ratio of the 4wt construct (Fig. 7c). In contrast to the VBS, elimination of the proton-binding site in a single subunit (3wt/qa construct) led to a dramatic reduction in proton-mediated TRPV1 activation (Figs 4 and 7). Moreover, a receptor lacking three of its four protons-putative subunits (wt/3qa) nearly lost its sensitivity to protons (Fig. 7b,c).

In order to further determine whether protons activate TRPV1 in a distinct stoichiometry as compared to vanilloids, we analyzed the concentration-response relationship of the concatemeric qa mutated constructs, using the whole-cell configuration of the patch clamp technique. Although the elimination of a single proton-binding site dramatically decreases the receptor maximal response to protons (Fig. 7b,c), receptor sensitivity and Hill coefficient were unaffected (4wt construct: EC_50_ = 5.23 ± 0.24, n_H_ = 1.8; 3wt/qa construct: EC_50_ = pH 5.20 ± 0.32, n_H_ = 1.6; Fig. 7d). Due to the impaired proton response in the 2wt/2qa and wt/3qa constructs, we were not able to obtain saturation responses and, therefore, could not determine the concentration-relation of these constructs. To conclude, our results indicate that full protons-mediated TRPV1 activation requires all four subunits.

## Discussion

Here we aimed to determine whether TRPV1 activation through different agonist binding sites follows distinct allosteric mechanisms. We combined the use of concatemeric constructs harboring mutated binding sites with patch-clamp recordings in order to determine the stoichiometry for TRPV1 activation through the vanilloid binding site (VBS) and the outer-pore domain by capsaicin and protons, respectively. Our results showed that while a single active VBS was sufficient to evoke full TRPV1 activation, similar proton-mediated channel activation required all four TRPV1 subunits. Thus, our findings demonstrated a distinct stoichiometry of TRPV1 activation through two of its different agonist-binding domains.

Interestingly, our results showed that not only a single active VBS was enough for capsaicin-mediated TRPV1 activation; it was sufficient to achieve a maximal open-channel lifetime (Figs 4 and 6). Thus, our findings suggest that the open channel stability is independent of the number of available binding sites, but rather depends on the duration of VBS occupancy. We further observed a shift in the capsaicin dose-response relationship as a direct function of the number of intact VBS (Fig. 4), indicating the presence of four functional binding sites in the wild-type TRPV1, and suggesting that the potency of capsaicin stems from its ability to independently bind each of the four sites. Capsaicin, similar to other exo- and endo-vanilloids, is a lipophilic molecule that needs to cross the plasma membrane in order to bind to VBS. Therefore, capsaicin efficacy derives from its potential accumulation within the cell and in proximity to the plasma membrane, in combination with the availability of four binding sites on its receptor. Relatively high capsaicin concentrations are required to achieve full TRPV1 activation through a single functional VBS (30 μM for wt/3ya vs. 2 μM for 4wt), which likely represents a scenario of binding sites occupancy rather than gating deficiency. Taken together, our results demonstrate that, while a single VBS is sufficient for capsaicin-mediated TRPV1 activation, high ligand potency requires the availability of all four VBS.

Similar to our findings regarding capsaicin-mediated activation of TRPV1, Janssens and Voets (2011) showed that TRPM8 harbors four independent binding sites for its agonist menthol^22^. However, contrary to our findings with TRPV1, their data demonstrates a stepwise mechanism for channel open state stabilization by the agonist. Therefore, although both somatosensory TRP channels have four independent binding sites for their respective lipophilic ligands (capsaicin for TRPV1 and menthol for TRPM8), their gating mechanisms appear to be different. Nevertheless, VBS-mediated TRPV1 activation follows a similar mechanism to that of the α7 AchR, shown by Andersen *et al.* (2013) to be fully activated through a single neurotransmitter-binding site^44^.

We obtained Hill slope factors of ~1 for all capsaicin-sensitive TRPV1 constructs (Figs 3 and 4). Various Hill coefficient values for capsaicin-mediated TRPV1 activation have been previously reported, their values generally in the 1–2 range^23^,^38^,^39^. Hill coefficient values are equal to the number of ligand binding sites, assuming the unlikely scenario that all bindings are completely cooperative^39^,^45^. Therefore, the obtained value potentially depends on the experimental procedures used in each study^46^.

In contrast to VBS/capsaicin-mediated TRPV1 activation, our results suggest that proton-mediated channel activation through its outer-pore domain behaves in a recessive manner (Fig. 7). Mutation of a single proton-binding subunit significantly reduced proton (pH 4)-evoked current (in whole-cell recordings at −40 mV), whereas mutating three subunits nearly abolished TRPV1 activation. We suggest that protonation of each subunit is needed for shifting the channel from its resting to active conformation, resulting in a recessive manner of proton-mediated TRPV1 activation.

An elegant, simplified model for separation of ligand-binding from activation gating of ligand-gated ion channels was recently summarized by William Zagotta (2015)^47^, based on a model originally proposed by Del Castillo and Katz (1957)^48^:


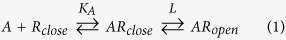


Where *A* is the agonist, *R* is the receptor, *K*_*A*_ is the association equilibrium constant for binding and *L* is the equilibrium constant of the bound channel. Our results suggest that while a single capsaicin-sensitive VBS (in the wt/3ya construct) is sufficient for full opening of the channel, maximal open probability of this construct requires high activator concentration (Figs 4 and 6). Hence, mutations in VBS mostly affected *K*_*A*_. On the other hand, while abolishing the proton-binding capability of one subunit (in the 3wt/qa construct) dramatically decreased the amplitude of the proton-evoked current, no shift in dose response was obtained (Fig. 7). Hence, mutations in the proton-binding site primarily affected *L*. Therefore, our results indicate that TRPV1 activation mechanism is activator-dependent.

## Methods

### TRPV1 Receptor Mutagenesis

The tetrameric TRPV1 channel was generated by linking the N- and C- termini of four rTRPV1 genes with a short linker sequence (-GSGGSGS-) and flanking them with distinct restriction sites relative to the MCS of pCDNA3.1+ (Invitrogen, MA, USA). The methionine of the first subunit and the stop codon of the last were conserved, while the remaining were removed. The concatemers were sub-cloned sequentially into pCDNA3.1+ (Invitrogen, MA, USA) using T4 DNA ligase (Thermo Scientific, MA, USA).

Site directed mutagenesis of Y511A was performed on the rTRPV1 gene through overlap PCR techniques using Phusion High-Fidelity DNA Polymerase (New England Biolabs, MA, USA), as previously described^12^,^49^. The restriction site AfeI was silently included with the mutation. The E600Q/E648A mutations were created using Q5 site mutagenesis kit (New England Biolabs, MA, USA) and silently included the restriction site BglII with the E648A primer. All constructs were verified by sequencing and restriction enzymes digestion.

### Cell culture and transfection

Human embryonic kidney 293 cells (HEK293T) were cultured in Dulbecco’s modified Eagle’s medium (DMEM) (Sigma-Aldrich, MO, USA) supplemented with 10% fetal bovine serum (FBS), 1% Penicillin-Streptomycin, 2 mM l-Alanyl l-Glutamine, and 25 mM HEPES (pH 7.3; Biological industries, Israel) (herein: Full DMEM) at 37 °C and 5% CO_2_. Cells were passed twice a week. Transfections were performed in 12-well plates containing 3 × 10^5^ cells using Mirus LT1 transfection reagent (Mirus Bio, WI, USA) with Opti-MEM I Reduced Serum Medium (Invitrogen, MA, USA). Generally, 300 ng of rTRPV1 in pCDNA3.1+ was used for transfection, while 700 ng of any of the concatemeric constructs expressed in pCDNA3.1+ were used. 200 ng EGFP inserted in pCDNA3.1+ was included with all DNA transfection to confirm successful transfection.

### Generating and inducing stably expressing Flp-in TREX 293 cells

All combinations of the Y511A mutation were stably expressed in Flp-in TREX 293 cells following manufacturer’s protocol (Invitrogen, MA, USA). Briefly, cells were co-transfected (performed as described above) with pcDNA5/FRT/TO containing the gene of interest and the Flp-recombinase expression vector pOG44 into the Flp-In host cell line (Invitrogen, MA, USA) at a ratio of 1:9, respectively, as previously described^12^. Successful recombination and maintenance of the gene of interest was confirmed through hygromycin B (200 μg/ml; Sigma-Aldrich, MO, USA) selection to establish a stably-transfected cell line.

### HEK293T Electrophysiology

Patch clamp experiments were executed using HEK293T cells in the whole cell or outside-out configuration 24 hrs after transfection, as previously described^12^,^49^,^50^,^51^. 30,000 cells were plated on glass coverslips (12 mm) coated with relevant amounts of poly-D-Lysine (PDL; Sigma-Aldrich, MO, USA) and/or Matrigel (BD-biosciences, MA, USA) according to the configuration (see below). Patch electrodes were fabricated from borosilicate glass using the P1000 Micropipette Puller (Sutter Instrument, CA, USA) and fire-polished using the microforge MF-900 (Narishige, Tokyo, Japan). Electrodes were coated with Sylgard (World Precision Instrument, FL, USA) to reduce the capacity of the recording pipette and to improve noise characteristics^52^. The bath solution contained (mM): 140 NaCl, 2.3 KCl, 2 MgSO 5 (4-(2-hydroxyethyl)-1-piperazineethanesulfonic acid (HEPES), and 5 2-(N-morpholino)ethanesulfonic acid (MES) and was adjusted to pH 7.4 with NaOH. Calcium was excluded from external solution in order to avoid TRPV1 calcium dependent desensitization^53^. Following establishment of the whole cell or outside-out configuration^36^, cells were perfused using the ValveBank perfusion system (AutoMate Scientific, CA, USA). Stock solution of 10 mM Capsaicin (Sigma-Aldrich, MO, USA and Tocris bioscience, Bristol, UK) was prepared in DMSO (Sigma-Aldrich, MO, USA), and freshly dissolved in bath solution to the desired concentration. Recordings were performed using Axopatch 200B patch clamp amplifier (Molecular Devices, CA, USA), and data was collected and analyzed using pClamp 10.2 software (Molecular Devices, CA, USA). All experiments were carried out at room temperature.

### Whole Cell Recordings

HEK293T cells were plated on coverslips pre-coated with 0.05 mg/ml poly-D-Lysine (PDL; Sigma-Aldrich, MO, USA) and 1 μg/ml Matrigel (BD-biosciences, Bedford, MA, USA) three hrs prior to recordings. Patch clamp experiments were filtered at 1 kHz using a low-pass Bessel filter and sampled at 2–5 kHz. Patch electrodes were fire-polished to a resistance of 2–4 MΩ. To avoid potential voltage errors due to receptor overexpression, dose responses were recorded at −40 mV, with the maximal current limited to ~2.5 nA. The pipette solution contained (mM): 100 Cesium methanesulfonate (CsMeSO_3_), 25 CsCl, 3 MgSO_4_, 3.62 CaCl_2_, 10 ethylene glycol tetraacetic acid (EGTA) and 30 HEPES adjusted to pH 7.2 with CsOH. Proton response recordings were performed with 50 μM Amiloride in the bath solution (Sigma-Aldrich, MO, USA) in order to block natively expressed ASICs^43^.

### Single Channel Recordings and Analysis

Single-channel currents were recorded in the outside-out patch configuration of the patch-clamp technique, as previously described^12^,^49^,^51^. All single-channel recordings were done using the stable cell line Flp-in TREX 293 cells. Cells were plated on coverslips coated with 0.2 mg/ml PDL one hour prior induction. Doxycycline concentration and induction times for obtaining a membrane patches with a single channel were determined for each construct^37^. Generally, 0.3–1 μg/ml doxycycline was used for 2–4 hrs prior to recordings. Patch electrodes were fire-polished to a resistance of 10–15 MΩ. The pipette solution contained (mM): 130 KCl, 4 NaCl, 2 MgSO_4_, 0.5 CaCl_2_, 1 EGTA and 10 HEPES adjusted to pH 7.2 with KOH. Single-channel currents were low-pass filtered at 5 kHz and digitized at 50 kHz. Occasional large brief noise spikes were visually identified and removed from the current traces. Single-channel recordings were analyzed with the pCLAMP 10.2 program Clampfit (Axon Instruments, USA), as previously described^12^,^51^. For each concatemeric construct, 150–2000 events were collected from each of 3–5 separate patches. To determine channel amplitude, all-point amplitude histograms were created using data digitally filtered at 2 kHz. To determine channel open probability, traces filtered at 5 kHz were idealized using the half-amplitude threshold crossing method (pCLAMP 10.2)^38^.

### Oocyte Preparation and Electrophysiology

Stage V–VI *Xenopus laevis* oocytes were prepared as previously described^12^,^49^. Oocytes were injected up to 24 hr after isolation with a total of 5 ng cRNA, and assayed 5–10 days later. Two electrode voltage-clamp recordings were carried out at RT using GeneClamp 500 connected to digidata1322A and pCLAMP 8.2 (Molecular Devices, CA, USA). Electrodes (Sutter Instruments, CA, USA) were filled with 3 M KCl and had resistance of 0.5–1 MΩ. Oocytes were continuously perfused with normal frog ringer (NFR) solution without calcium containing (mM): 115 NaCl, 2.5 KCl, 2 MgSO_2_, and 10 HEPES, pH 7.4 with NaOH.

### Western Blot Analysis

For Western-blot analysis under reducing conditions, HEK293T cells were transiently transfected with 4 μg of concatemeric rTRPV1 (4wt), 1.5 μg of wild type TRPV1, or 2.5 μg of EGFP (all in pCDNA3.1+) in 6-well plates with 7.5 × 10^5^ cells, as previously described^49^,^50^. The following day cells were washed with ice cold PBS containing 0.2 g/L KH_2_PO_4_, 0.2 g/L KCl, 8.0 g/L NaCl, and 1.15 g/L Na_2_HPO_4_ (Sigma-Aldrich, MO, USA), and then mechanically lifted. The samples were centrifuged for 30 s at 1000xg and then resuspended in a RIPA solution containing 150 mM NaCl, 1.0% IGEPAL® CA-630, 0.5% sodium deoxycholate, 0.1% SDS, and 50 mM Tris, pH 8.0 and protease inhibitors including 1 mM AEBSF, 800 nM Aprotinin, 50 μM Bestatin, 15 μM E64, 20 μM Leupeptin, and 10 μM Pepstatin A, dissolved in DMSO (all regents from Thermo Scientific, MA, USA). Samples were incubated on ice for 30 min, with gentle perturbation every 10 min. After centrifuging for 30 min, the supernatant (total protein extract) was collected.

For each 10 μl of total sample volume, a ratio of 6.5 μl of lysis sample, 2.5 μl of Lithium Dodecyl Sulfate (LDS) sample buffer, and 1 μl of the reducing agent Dithiothreitol (DTT) (Invitrogen, MA, USA) were combined and then boiled at 95 °C for 5 min. Due to the predicted size of the concatemeric constructs (~400 kDa), protein samples were separated on a 3–8% SDS-PAGE Tris-Acetate gradient gel and transferred to a nitrocellulose membrane (Invitrogen, MA, USA) in the presence of 5% transfer buffer (Invitrogen, MA, USA), 0.1% Antioxidant (N,M-Dimethylformarmide), and 5% Methanol in double distilled water. The membrane was blocked with 5% nonfat dry milk in Tris-buffered saline (TBST) containing 50 mM Tris, 150 mM NaCl and 0.05% Tween 20. The membrane was incubated with primary rabbit-anti-rat rTRPV1 antibodies dissolved in TBST (1:2000; Alomone Labs, Jerusalem, Israel)^54^ overnight at 4 °C with gentle agitation. Blots were washed three times for 5 min each and incubated with goat-anti-rabbit HRP conjugated antibody (1:10,000; Jackson ImmunoResearch, PA, USA) for 1 hr at RT, followed by developing with an ECL kit (PerkinElmer, MA, USA). Images were acquired using ChemiDoc XRS+ molecular imager (Bio-Rad, CA, USA) and analyzed using ImageLab software (Bio-Rad, CA, USA).

### Data Analysis

Dose response curves were calculated by SigmaPlot software (San Jose, CA) using the sigmoidal Hill equation as follows:


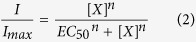


Where: *I* = measured current, *I*_*max*_ = maximal current at saturating dose (pre-measured for each construct), *x* = tested agonist concentration, *EC*_*50*_ = the calculated concentration that elicits 50% of maximal current and *n* = Hill coefficient.

Thermal threshold was determined by normalizing the response at each temperature to the response at the maximal applied temperature (50 °C; holding potential of +80 mV)^55^. Temperature thresholds represent the point of intersection between linear fits and baseline and the steepest component of the Arrhenius profile. Arrhenius curves were obtained by plotting obtained current on a log-scale against the absolute temperatures. *Q*_*10*_ was calculated using the following equation:


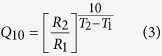


Where *R*_*2*_ = current at the higher temperature (*T*_*2*_) and *R*_*1*_ = current at the lower temperature (*T*_*1*_).

Standard errors were calculated using SigmaPlot software (San Jose, CA). Student’s *t*-test was used to determine statistical similarities between different groups.

## Additional Information

**How to cite this article**: Hazan, A. *et al.* The pain receptor TRPV1 displays agonist-dependent activation stoichiometry. *Sci. Rep.* **5**, 12278; doi: 10.1038/srep12278 (2015).
